# Vitamin D, gut microbiota, and radiation-related resistance: a love-hate triangle

**DOI:** 10.1186/s13046-019-1499-y

**Published:** 2019-12-16

**Authors:** Ruixue Huang, Jing Xiang, Pingkun Zhou

**Affiliations:** 1 Department of Occupational and Environmental Health, Xiangya School of Public Health, Central South University, Changsha, 410078 Hunan Province China; 2 Institute for Chemical Carcinogenesis, State Key Laboratory of Respiratory, School of Public Health, Guangzhou Medical University, Guangzhou, 511436 People’s Republic of China; 3 Department of Radiation Biology, Beijing Key Laboratory for Radiobiology, Beijing Institute of Radiation Medicine, AMMS, Beijing, 100850 China

**Keywords:** Radiation resistance, Gut microbiota, Vitamin D

## Abstract

Radiation resistance is a serious issue in radiotherapy. Increasing evidence indicates that the human gut microbiome plays a role in the development of radiation resistance. Vitamin D is an important supplement for cancer patients treated with radiotherapy. Against this background, this paper reviewed research regarding the associations among vitamin D, microbiota dysbiosis, and radiation resistance. A hypothesis is developed to describe the relationships among vitamin D, the gut microbiota, and radiotherapy outcomes. Radiotherapy changes the composition of the gut microbiota, which in turn influence the serum level of vitamin D, and its distribution and metabolism in the body. Alteration of vitamin D level influences the patient response to radiotherapy, where the underlying mechanisms may be associated with the intestinal microenvironment, immune molecules in the intestines, gut microbiome metabolites, and signaling pathways associated with vitamin D receptors. Our understanding of the contribution of vitamin D and the gut microbiota to radiotherapy outcomes has been increasing gradually. A better understanding of the relationships among vitamin D, the gut microbiota, and radiotherapy outcomes will shed more light on radiation resistance, and also promote the development of new strategies for overcoming it, thus addressing an important challenge associated with the currently available radiotherapy modalities for cancer patients.

## Background

In 2017, the global death population caused by cancer reached 9 million, which was nearly twice the number in 1990 [1]. In 2018, 18.1 million new cancer cases, and 9.6 million deaths from cancer, were reported worldwide [2]. Numerous treatments are available for non-melanoma skin cancer patients, with radiotherapy being an efficacious and tissue-preserving non-surgical option [3]. Radiotherapy is defined as the clinical use of ionizing radiation (IR), including α or γ rays, to induce DNA damage in all exposed cells to ultimately kill cancer cells or prevent cancer growth [4, 5]. It can be used to eradicate certain cancers or reduce their likelihood of recurrence, and as a palliative treatment [6]. Currently, approximately 60% of patients being treated for cancer in the United States have received radiotherapy. Despite the increasing clinical application of radiotherapy, the resistance of tumor cells to IR remains a significant obstacle [7], potentially leading to relapse, a poor treatment response or poor prognosis [8–13]. Moreover, radiation resistance induces injury to tumor-adjacent tissues, resulting in disruption of normal physiological functions, as expressed in symptoms such as diarrhea and rectal bleeding [14], and significantly increasing the subsequent risk of a number of adverse events including cardiovascular disorders, micronutrient deficiencies, and even secondary tumors, all of which typically decrease patient quality of life. The phenomenon of radiation resistance presents two challenges to the advancement of radiotherapy: (1) development of a mechanistic understanding of the factors underlying radiation resistance and the heterogeneity thereof; and (2) development of effective treatments, based on clinical and experimental molecular methods, to decrease side effects and overcome radiation resistance in cancer patients. Currently, despite the rapid development of new technologies, our understanding of, and ability to treat, cancer is still limited by many factors, including radiation resistance [15]. The current perspective on resistance mechanisms, which is a complex process involving multiple genes, factors, and signaling pathways, points to an unmet need to examine novel factors, including the functional role of the gut microbiota. Additionally, utilizing a nutrient-focused approach in individual cancer patients may improve the likelihood of successful radiotherapy, a reduced rate of side effects, and long-lasting benefits. Against this background, this review discusses areas of mechanistic understanding that may benefit from a new perspective.

### Radiation resistance-related mechanisms

**Fig. 1:**
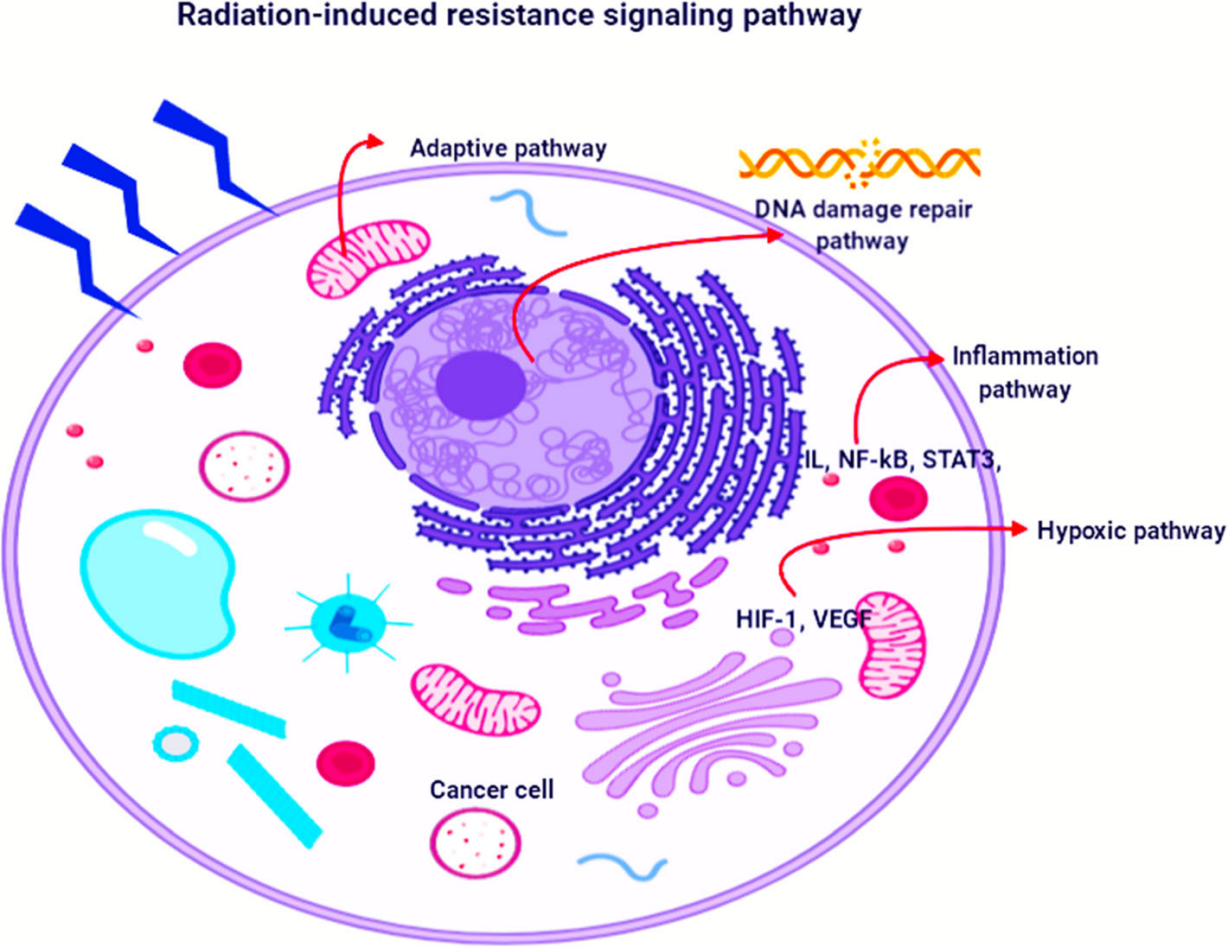
Signaling pathways involved into the radiation-induced resistance. The extensive studied signaling pathways consist of DNA damage repair pathway, inflammation pathway, hypoxic pathway

Ionizing radiation deposits energy and generates reactive chemical species along “tracks”, resulting in cytotoxic and genotoxic injury to DNA, including DNA double-strand breaks, and posing a challenge to cancer cell survival by inhibiting the proliferation thereof [16]. Additionally, IR can induce cell cycle arrest, apoptosis, autophagy, and changes in the cellular microenvironment [12, 17], which can in turn lead to radiation resistance. Cancer cells may develop mechanisms to escape cell cycle arrest, resist DNA damage-induced cell apoptosis, or alter the cancer microenvironment through cytokines [18–20]. The radiation resistance associated with these changes benefits cancer cells and renders radiotherapy less effective. Several signaling pathways contribute to cellular resistance against IR (Fig. 1) [21]. Although radiation resistance-related molecular mechanisms have been intensively investigated, many questions remain unresolved. For example, does crosstalk exist between individual mechanisms, and are there genes and proteins important in multiple mechanisms? For example, some reports have identified genes and proteins involved in both IR-induced cell cycle checkpoints and autophagy [22]. Are there other mechanisms that must be considered for a complete understanding of radiation resistance? Moreover, is there a single core regulator of multiple radiation resistance-related signaling pathways, active during all radiation resistance processes? [23, 24].

### Association between radiotherapy and gut microbiota

The gut microbiota, i.e., the bacteria, archaea, viruses, and eukaryotic microbes residing primarily in the colon (but also in other organs including the lung and stomach) [25], accounts for approximately 1 kg of human body weight, and includes more unique genes than the human genome [26]. Over the past decade, rapid development of DNA and 16 s RNA sequencing technology has dramatically improved researchers’ ability to survey changes in gut microbiota in response to different stresses [27]. Increasingly, reports have indicated that the gut microbiota plays a major role in the maintenance not just of intestinal homoeostasis, but of the overall health of the body [28]. In particular, the dysbiotic gut microbiome seen in cancer radiotherapy patients, with altered microbial diversity and richness relative to that of healthy individuals, has been associated with the outcomes of cancer therapy [29]. Recently, it has been shown that gut microbiota status is closely related to the response to radiotherapy. Many studies have discussed the effectiveness of radiotherapy for various types of cancer, and radiotherapy-related side effects, in the context of the gut microbiota. Tilg et al. recently reported a direct link between altered microbiota composition and the inflammatory status of patients with type 2 diabetes; decreased diversity of the intestinal microbiota may lead to a failure to maintain the intestinal barrier needed to prevent systemic dissemination of gut bacteria and associated chemical mediators [30]. Daily ultraviolet radiation of skin is a typical source of 25-hydroxyvitamin D3 (25(OH)D3) in the human body. Previous studies reported that ultraviolet radiation was associated with a significant change in the beta-diversity of feces. Specifically, members of the phylum Firmicutes family, including *Coprococcus*, were enriched, whereas members of the phylum Bacteroidetes family, such as Bacteroidales, were depleted [31].

During the process of radiotherapy, gut microbiota including *Lactobacillus acidophilus*, *L.casei*, and *Bifidobacterium* spp. have been proven to reduce symptoms of radiation-induced gut toxicity, such as diarrhea [27]. However, Barker et al.(2015)reported that radiotherapy altered the composition of the gut microbiota, breaking the intestinal barrier and causing apoptosis in intestinal crypts [32], although other studies found no effect of radiation on the gut microbiota. For instance, Gosiewski et al. showed that therapeutic doses of radiation did not significantly affect *Lactobacillus* populations [33]. Dysbiotic gut microbiome may be due to factors other than irradiation, such as heterogeneity among patients, including with respect to their daily diets, alcohol intake, and medication use. This highlights the challenges faced by studies of the association of the gut microbiota with radiation resistance seeking to answer the following important questions. Which taxa/phyla play the most dominant role in the development of radiation resistance? How are gut microbiota-derived signaling molecules generated, and how do they increase or suppress radiation sensitivity? How do factors such as nutrients induce gut microbiota changes, and how is this linked to radiation resistance?

### Vitamin D: role in biological processes

Vitamin D comprises a group of fat-soluble secosteroids responsible for the absorption of essential trace elements, such as calcium, magnesium, and phosphate, and having roles inmultiple biological processes [34], including cell growth, as well as in immune function and inflammation (reduction thereof). Vitamin D supplements are provided to treat or prevent many diseases, including deficiency-induced rickets and osteomalacia [35, 36]. For example, clinically, the high incidence and poor prognosis of colorectal cancer has been found to be partly attributed to insufficient vitamin D [37], and colorectal cancer patients with high levels of vitamin D have a lower risk of metastatic progression during neoadjuvant therapy before radical surgery [37]. A meta-analysis by Van den Blink et al. reported that vitamin supplementation reduced radiation-related bone fractures and the risk of avascular necrosis in patients undergoing pelvic radiotherapy [38]. Castro-Equiluz et al. recommended vitamin D as the most important nutrient for cancer patients treated with pelvic radiotherapy [14]. Typically, the natural form of cholecalciferol is produced in the skin from dehydrocholesterol, with pre-vitamin D3 produced after ultraviolet irradiation. This process is essential for vitamin D biosynthesis in humans, although vitamin D can also be supplied via the diet. In the body, vitamin D is transported into the blood and metabolized in the liver, where it is then hydroxylated to produce the active form, 25-hydroxyvitamin D3 (25(OH)D3). Many cytochrome P-450 enzymes are involved in the conversion of vitamin D to 25(OH)D3, including CYP2R1, CYP27A1, and CYP2D25 [39]. This active form of vitamin D has numerous biological effects, including inhibition of the epithelial-mesenchymal (EMT) transition in cancer cells; it also confers protection against cardiovascular disease and inflammatory bowel disease. 25(OH)D3 prevents the EMT in human peritoneal mesothelial cells through regulation of the Wnt/β-catenin signaling pathway [40]. Hou et al. observed that 1α, 25(OH)2D3 suppressed the migration of ovarian cancer cells by inhibiting the EMT, suggesting that1α,25(OH)2D3 might have potential as a therapeutic agent for ovarian cancer [41]. Furthermore, Findlay et al. showed that 1α,25(OH)2D3 enhanced radiation sensitivity in colorectal cancer cells through regulating the EMT [42]. Higher plasma levels of 25(OH)D3 are associated with a decreased risk of highly aggressive prostate cancer [43]. Mutation or deficiency of the genes and enzymes responsible for the transport or metabolism of 25(OH)D3 may alter its levels and functions [43]. For instance, a mutation in CYP2R1, a key hydroxylase for 25(OH)D3 production, resulted in deficiency thereof, as well as symptoms of vitamin D-dependent rickets [44]. In addition to its classic effects on calcium and bone homeostasis, vitamin D has other important roles in immune regulation and protection of the cardiovascular system [45]. As reported in the review article by Aranow, vitamin D receptors are expressed on immune cells, including B cells, T cells, and antigen-presenting cells; this indicates that active vitamin D metabolites are synthesized by these cells, suggesting that vitamin D can modulate innate and adaptive immune responses. In turn, this suggests that the beneficial effects of vitamin D supplementation in deficient individuals with autoimmune disease may extend beyond the effects on bone and calcium homeostasis [45]. Similarly, vitamin D has a putative protective role in the cardiovascular system [46]. Growing evidence suggests that vitamin D levels are inversely associated with the risk of cardiovascular diseases, including ischemic heart disease, stroke, hypertension, blood lipids abnormalities, and obesity [47]. However, some studies, including randomized controlled trials (RCTs), did not report significant effects of vitamin D supplementation on cardiovascular outcomes [48], suggesting a need for further research. Current evidence indicates that vitamin D plays important roles in cardiovascular function, but more data are needed to establish causality. In this review, we focused on the underlying mechanisms of radiation resistance; thus, in the discussion below, we will address the following: (i) the association of vitamin D level with the risk of radiotherapy-induced side effects; (ii) the association of vitamin D level with radiation resistance; (iii) the effects of vitamin D on the integrity of the intestinal barrier, (iv) the effects of vitamin D on the gut microbiota; and (v) the effects of gut microbiota on vitamin D metabolism, distribution, and utilization.

### Vitamin D-mediated roles in radiation resistance

Recently, there has been increasing concern regarding the role of vitamin D in preventing radiotherapy-induced side effects. Surrounding tissues may be damaged during radiotherapy, leading to IR-induced symptom including diarrhea and rectal bleeding [14]. A study by Mukai et al. indicated that vitamin D supplementation was a significant factor in prolonged metastasis-free survival after preoperative chemoradiation therapy for patients with pancreatic ductal adenocarcinoma [49]. Radiation dermatitis occurs frequently during radiation therapy in cancer patients, and vitamin D ointment is helpful for its prevention [50]. In a case report, vitamin D supplementation prior to surgery and radiotherapy in a patient with recurrent breast cancer altered certain biological cancer markers, such as estrogen receptor, human epidermal growth factor receptor, and nuclear protein Ki67 [51]. Moreover, an increasing body of evidence suggests that gut epithelial vitamin D receptor signaling pathways play an essential role in maintaining the integrity of the intestinal mucosa. Vitamin D deficiency is associated with the severity of radiation-induced proctitis in cancer patients [52]. However, the mechanisms underlying the ability of vitamin D to decrease radiotherapy-induced side effects needs to be elucidated so that appropriate management guidelines and recommendations for cancer patients undergoing radiotherapy can be formulated. Sharma et al. found that 25(OH)D3, the hormonally active form of vitamin D [53], promoted responses of non-small cell lung cancer to irradiation through induction of autophagy via the vitamin D receptor/TP53/AMPK signaling pathway [54]. Another study asserted that vitamin D has the potential to improve genetic inhibition and increase sensitivity to radiation, by acting as a switch between cytoprotective and cytotoxic autophagy [55]. Elegant studies have indicated that loss of the DNA repair protein 53BP1 results in resistance of breast cancer cells to radiation. The active form of vitamin D, 1α,25(OH)2D3, stabilizes 53BP1 levels in tumor cells, restoring them as efficiently as cathepsin L inhibitors, and ultimately contributing to increased genomic instability in response to radiation and reduced proliferation of cancer cells [56].

**Fig. 2:**
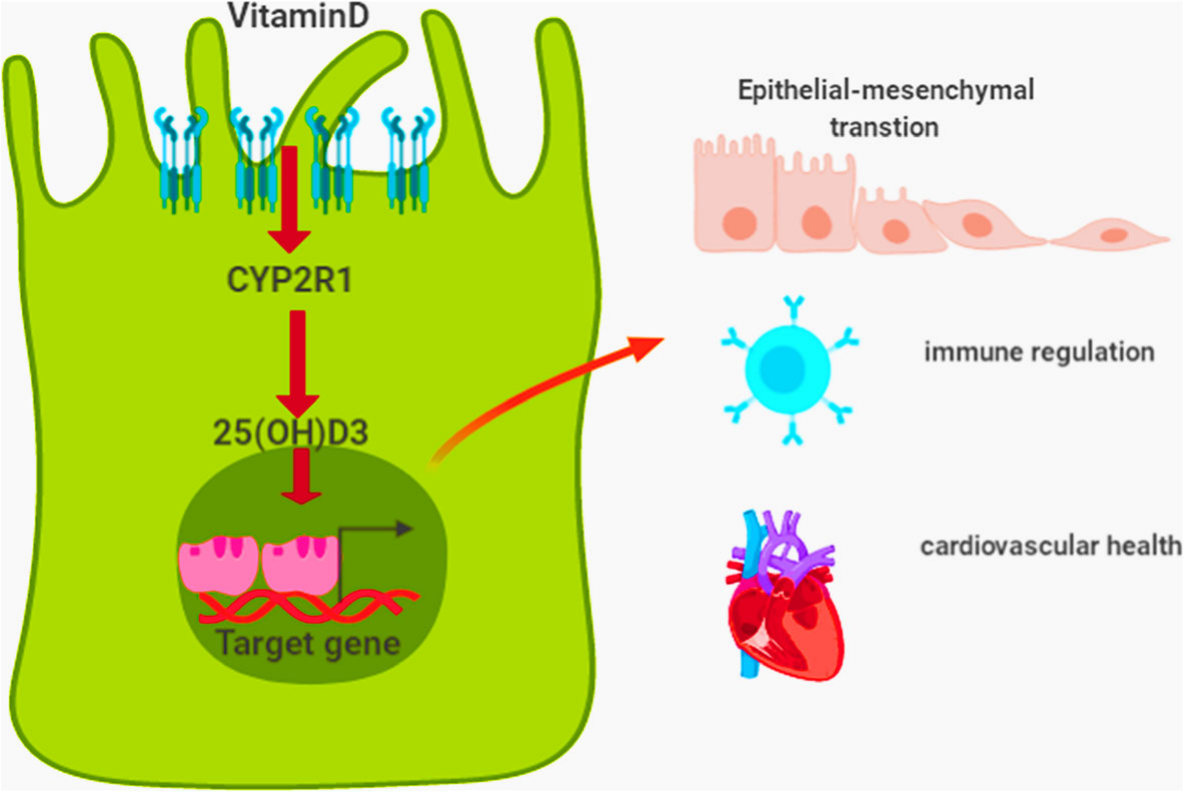
The molecular mechanism of Vitamin D’s role in the biological functions. 25(OH)D3, the major metabolite of vitamin D, binds vitamin receptors on intestinal cells and regulates the transcription of target genes, promoting gut health by maintaining immune homeostasis and suppressing inflammation and fibrosis

Strikingly, 25(OH)D3 is crucial for maintaining the intestinal barrier [57]. The physical intestinal barrier, comprised of a thick mucus layer and the epithelium, plays a critical role in the defense against microbes, harmful foreign antigens, endotoxins, and toxic metabolites of bacteria, in addition to other environmental hazards entering the body via the diet. The importance of 25(OH)D3 in the gut has been demonstrated over the past decade. In brief, 25(OH)D3 binds vitamin receptors on intestinal cells and regulates the transcription of target genes, promoting gut health by maintaining immune homeostasis and suppressing inflammation and fibrosis (Fig. 2). Furthermore, decreased intestinal epithelial vitamin D receptor expression alters gut microbial homeostasis, resulting in less butyrate production and, by extension, chemically induced colitis in mice [58]. In human studies, high-dose vitamin D_3_ supplementation had a beneficial effect on the human gut microbiota, markedly reducing typical opportunistic pathogens and increasing phylotype richness [59]. Butyrate, a byproduct of carbohydrate breakdown by microbiota, has a well-established role in preventing mucosal inflammation. Sun et al. showed that decreased expression of intestinal epithelial vitamin D receptors led to lower butyrate production and intestinal barrier inflammation [60]. Vitamin D protects the intestinal barrier by regulating tight junction proteins and inhibiting intestinal apoptosis [61]. Furthermore, vitamin D enhances innate immunity by inducing antimicrobial peptides, and regulates adaptive immunity by promoting anti-inflammatory T cells and cytokines [61]. A review by Cantorna et al. suggested that vitamin D deficiency increases susceptibility to infection or injury of the gastrointestinal tract [62]. Vitamin D enhances the ability of innate lymphoid cells to produce IL-22, suppresses IFN-γ and IL-17 release from T cells, and induces regulation of T cells in the mucosal tissues, modulating microbial communities in the gut to maintain the integrity of the intestinal barrier. Mandle et al. showed that vitamin D3 (1000 IU per day) significantly improved intestinal barrier function-related biomarkers, such as tight junction proteins claudin-1 (CLDN1), occludin (OCLD), and mucin-12 (MUC12), in patients with recurrent colorectal adenoma [63]. Disruption of intestinal epithelial barrier homeostasis typically occurs due to altered composition of the gut microbiota [64], and the interaction between vitamin D and the gut microbiota serves as a primary defense against radiation resistance.

Radiotherapy influences vitamin D levels. A recent gene expression study showed that mice exposed to IR exhibited lower expression levels of the CYP genes Cyp4f18 and Cyp4v3 [65]. CYP4 proteins have been reported to metabolize vitamin D and play an essential role in the defense against environmental stressors, including radiation exposure [66]. The involvement of vitamin D metabolism in radiation injury has been documented in several gene expression studies [67]. The vitamin D metabolite calcitroic acid increased in mice after high dose rate (HDR) cesium-137 (137Cs) and strontium-90 (90Sr) (1.1 Gy/min to) [67], whereas a low dose rate (LDR) (3.0 mGy/min) had no effect on this metabolite; this suggested that the effects on vitamin D metabolism differ by exposure level. However, studies in this area are limited, particularly those examining how radiotherapy influences the mechanisms of vitamin D metabolism.

### Effects of vitamin D-on the gut microbiota

In addition to protecting the intestinal barrier, vitamin D may favorably alter the gut microbiota [60], with evidence also emerging of its role in reducing the resistance of cancer cellsto radiation. In some human studies, high-dose vitamin D3 supplementation had a beneficial effect on human gut microbiota, markedly reducing typical opportunistic pathogen species including *Pseudomonas, Escherichia,* and *Shigella,* and increasing phylotype richness [59]. An association between vitamin D and radiation resistance has also been posited, via alteration of the gut microbiota. Ferrer-Mayorga et al. showed that the vitamin D metabolite 1α,25-dihydroxyvitamin D3 inhibits colorectal cancer cell proliferation and promotes epithelial differentiation of colon cancer cell lines, thereby improving radiation sensitivity through altering the composition of intestinal microbiota communities [68]. Some studies reported that vitamin D influenced the gut microbiome through activation of enteric bacteria vitamin D receptor signaling [69, 70]. Commensal and pathogenic bacteria directly regulate colonic epithelial vitamin D receptor expression, which in turn negatively regulates bacteria-induced intestinal nuclear factor-kappa B activation [71, 72]. Accordingly, vitamin D receptor gene mutations in humans should influence the intestinal microbiota. In vitamin D receptor knockout mice, *Parabacteroides* abundance was altered significantly [73], *Lactobacillus* was depleted, and *Clostridium* and *Bacteroides* showed enrichment [74]. Furthermore, vitamin D deficiency induces notable changes in the gut microbiota, including increased *Helicobacter hepaticus* and decreased *Akkermansiamuciniphila* population sizes [75]. *Lactobacillus sakei* is known to have a radioprotective effect for the enteritis compared to conventional chemical agents with inherent toxicities [76]. Intaking synbiotic powder containing *Lactobacillus reuteri* (108 CFU) reduce proctitis symptoms and improve quality of life by preventing rectal inflammation during radiotherapy for prostate cancer [77]. Moreover, *Bacteroides* increased in radiation-exposed conventional microbiota, and *H. hepaticus* is known to induce colon cancer [78]. *A. muciniphila* is also known to improve barrier function and metabolic health [79]. These data indicate that one mechanism via which vitamin D protects against radiation resistance is through targeting the gut microbiota via the vitamin D receptor. Moreover, alterations of gut microbiota can be caused by vitamin D intake and other dietary components, rendering gut microbiota regulation by vitamin D complex. Further study is needed to uncover and confirm the mechanisms underlying the effects of vitamin Don the gut microbiota.

Logically, altered gut microbiota should influence the vitamin D distribution and metabolism in the body. However, studies on the contribution of altered gut microbiota to these parameters are scarce. Bora et al. showed that germ-free mice infected with the pathogen *C. rodentium* exhibited decreased vitamin D and 25D absorption post infection [80]. They also measured serum 25-hydroxyvitamin D, 24,25-dihydroxyvitamin D, and 1,25-dihydroxyvitamin D levels before and 2 weeks after broad-spectrum antibiotic treatment; the levels of all three compounds were increased, which was attributed to the microbiota or antibiotic treatment [81]. A recent study examined the effect of the gut microbiota on vitamin D metabolism [80], and found that it inhibited fibroblast growth factor 23 and induced increased serum 25-hydroxyvitamin D, 24,25-dihydroxyvitamin D, and 1,25-dihydroxyvitamin D levels. Some researchers have suggested that different gut microbiota signatures and alterations in vitamin D3 levels may be useful markers of disease in clinical practice, and that fecal vitamin D3 and gut microbiota composition could serve as biomarkers for diagnosis and follow-up [82]. However, for effective interventions targeting the gut microbiome composition and vitamin D levels, further investigation is required, to determine the mechanisms underlying vitamin D regulation by the microbiota. Future studies should address the following questions. (i) What are the underlying mechanisms by which vitamin D regulates radiation resistance?; (ii) Are vitamin D levels regulated predominantly by one bacterial species, or by multiple, interacting species?; (iii) Environmental factors, including ultraviolet radiation, and lifestyle factors including reduced physical activity and insufficient consumption of vitamin D-rich foods, are involved in the alteration of gut microbiota and the etiology of vitamin D deficiency, and may also be important in radiotherapy outcomes; do these factors affect radiation resistance, and if so, how?

There is a pressing need for further investigation of the relationships among environmental factors, lifestyle factors, vitamin D levels, gut microbiota, and radiation resistance, to promote the development of vitamin D-based clinical interventions targeting the microbiota for addressing radiation resistance.

### Relationships among vitamin D, gut microbiota, and radiation resistance

**Fig. 3:**
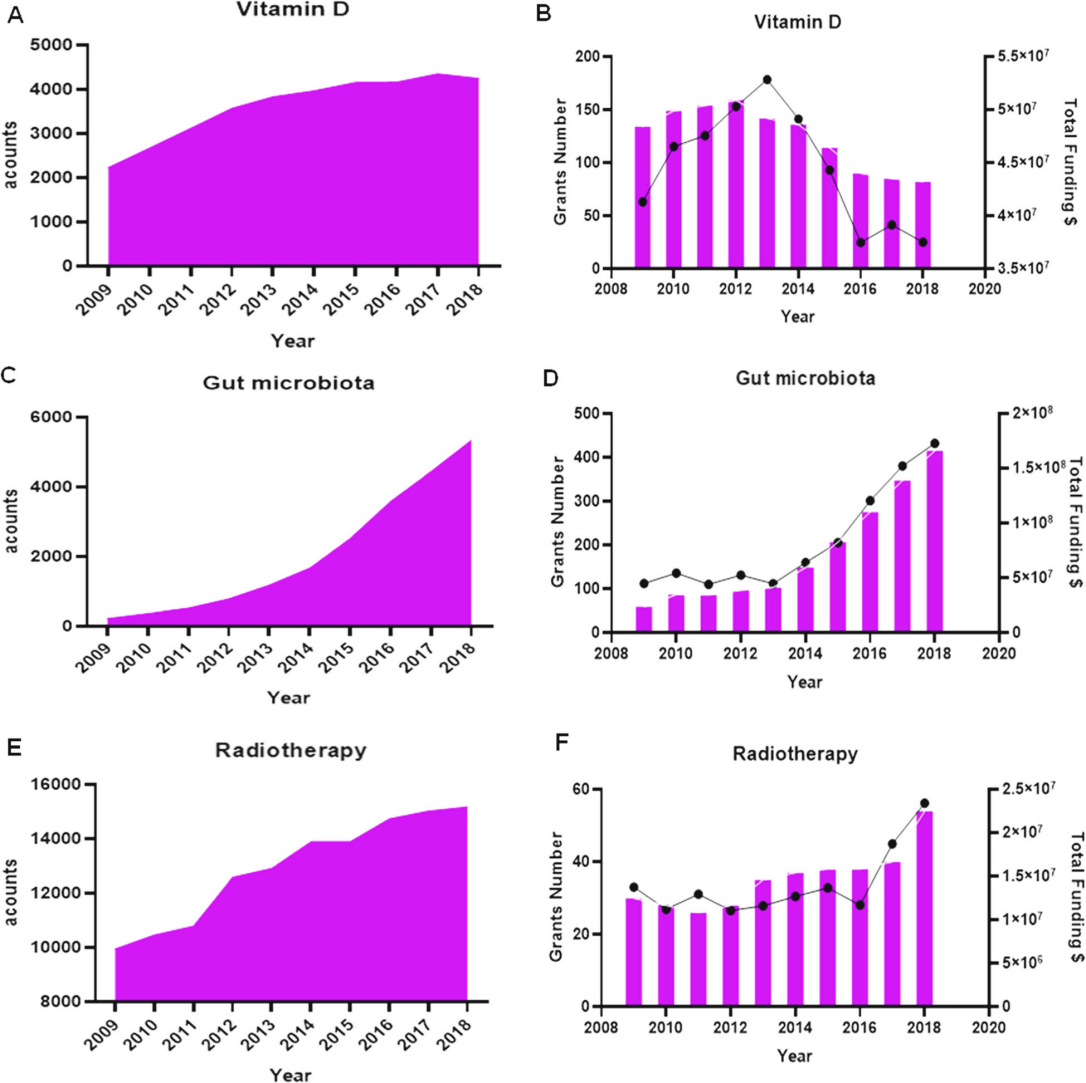
Study attention and grants trends on the vitamin D, gut microbiota and radiation-induced resistance from 2009 to 2018. (**a**) literatures amount of study attention on the field of vitamin D. (**b**) grants number and funding amounts of vitamin D. (**c**). literatures amount of study attention on the field of gut microbiota. (**d**) grants number and funding amounts of gut microbiota. (**e**) literatures amount of study attention on the field of radiotherapy. (**f**) grants number and funding amounts of radiotherapy

To review the research on the relationships of vitamin D, gut microbiota, and radiotherapy outcomes as well as acknowledge the study trend regarding these three topics, we searched published literature by the Pubmed(www.pubmed.com) and grants funded by the US National Institutes of Health (NIH, https://www.nih.gov/) over the past decade. We selected both two websites since Pubmed includes almost the possible published studies as possible as it can across the world scientific community, while NIH provide the grants funded mainly from developed counties such as USA and other developing countries such as India and China. As shown in Fig. 3, between 2009 and 2018, the number of publications on vitamin D, gut microbiota, and radiotherapy gradually increased, although grants and funding for vitamin D research have been gradually decreasing; in each of the last 3 years, there were fewer than 100 grants (Fig. 3a,b). In contrast, grants and funding for gut microbiota and radiotherapy research have been increasing gradually each year, and peaked in 2018(Fig. 3c,d). The total funding for gut microbiota research in 2018 was almost $200,000,000, far greater than that for vitamin D ($40,000,000) and radiotherapy ($25,000,000) research (Fig. 3e,f). Overall, these data suggest that vitamin D, gut microbiota, and radiotherapy are receiving attention from both the scientific community and governments.

**Fig. 4:**
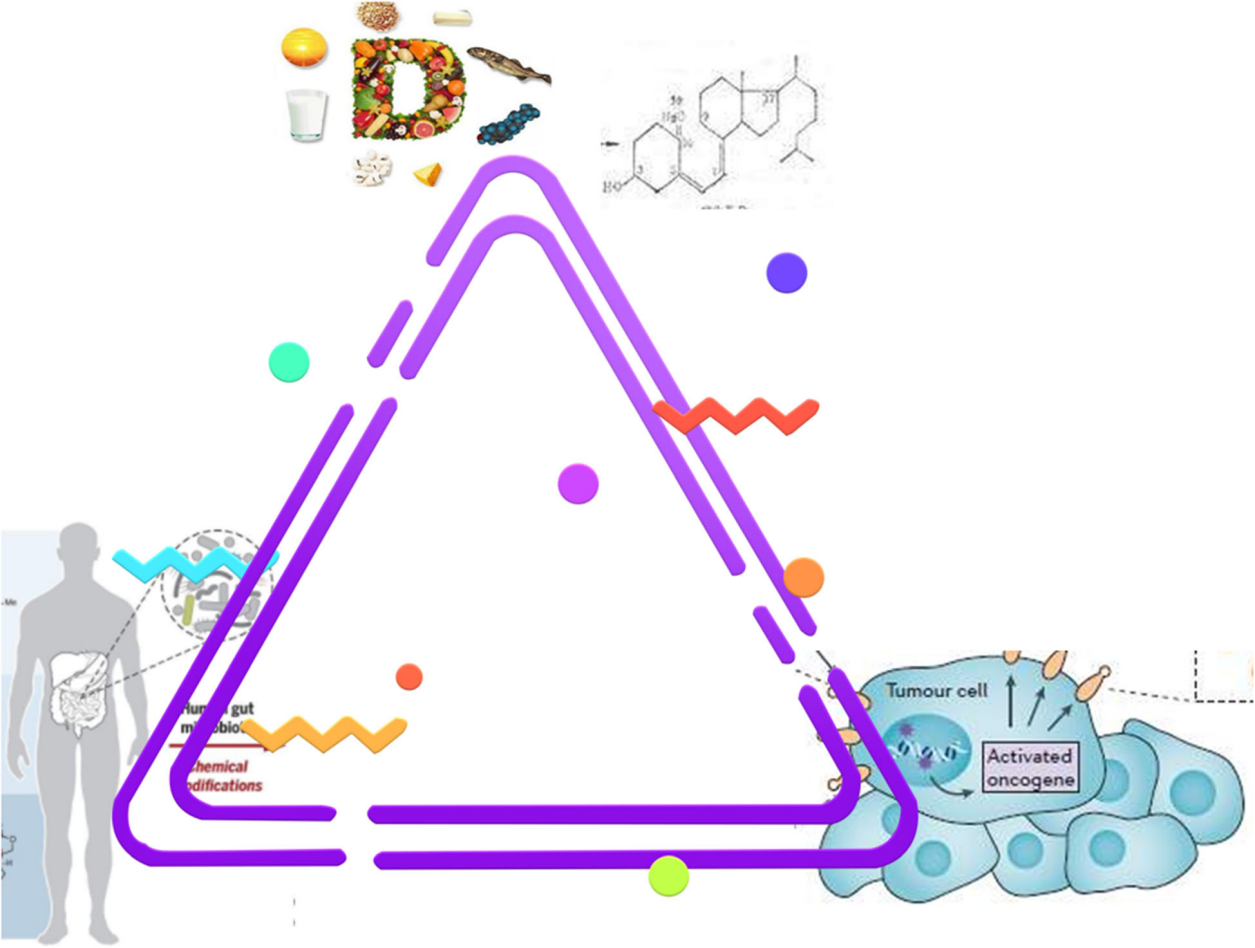
A triangle of the relationships among vitamin D, gut microbiota, and radiation-induced resistance

In summary, the relationships among vitamin D, gut microbiota, and radiotherapy outcomes can be described as a triangle, as illustrated in Fig. 4; the roles of the elements of this “love-hate triangle” differ according to the physiological/pathological status of the cell. Vitamin D plays a crucial role in protecting the intestinal barrier and preventing gastrointestinal mucosal inflammation. Vitamin D deficiency not only affects the integrity of the barrier, but also moderates the composition of the gut microbiome community in murine models. In humans, vitamin D deficiency, accompanied by vitamin D receptor gene mutations, also contributes to changes in the gut microbiome. Moreover, gut microbiota status influences vitamin D distribution and metabolism. Alterations of the gut microbiota have also been studied in relation to radiotherapy. Some species of gut microbiota are associated with radiation resistance, while radiation can in turn influence the gut microbiota composition, where marked changes are frequently seen in *Bifidobacterium*, *Clostridium*, and *Bacteroides spp*. Radiotherapy affects vitamin D metabolism and distribution in the body, which in turn affects radiotherapy outcomes; there are vitamin D receptor polymorphisms having differential sensitivity to radiation.

The interactions among vitamin D, the gut microbiota, and radiotherapy outcomes are important for understanding radiation resistance. Substantial progress has been made in our understanding of these interactions at the molecular level, which could help to guide strategies aimed at overcoming radiation resistance in radiotherapy patients. Nevertheless, many questions remain, as follows. (i) How and to what extent do distinct molecular pathways lead to a pathological imbalance in the “love-hate triangle”?; (ii) Does an unstable gut microbiome lead to progressive dysregulation of vitamin D metabolism beyond a critical threshold for radiotherapy-induced radiation resistance?; (iii) What other dietary factors and microbiome metabolites are associated with host responses to radiotherapy, and through what molecular receptors and signaling pathways do they interact with vitamin D?; and (iv) Although gut microbiota transplantation has been documented in numerous studies, with encouraging outcomes, their clinical applications are limited. Could such transplantations serve as a novel intervention in radiation resistance?

These questions are of great importance, given the fundamental challenges that remain with respect to overcoming radiation resistance and improving the quality of life of cancer patients. Obtaining answers to these questions will allow us to better understand the interactions among vitamin D, the gut microbiota, and radiotherapy outcomes, and could guide the development of new interventions to restore homeostasis in both the intestinal barrier and the microbiome.

## Conclusion

In conclusion, vitamin D and gut microbiota are key factors in shaping the radiation-induced resistance, and therefore, their impact on quality of patients’ life and cancer recurrence. However, the challenge now is to fully decipher the molecular mechanisms that link the vitamin D, gut microbiota, radiation resistance in a network of communication that impacts radiotherapy outcomes, to eventually translate these findings to the clinical prevention and control of radiation-induced resistance. Additional studies including measuring novel vitamin D metabolites by gut microbiota and utilizing randomized controlled trial to determine the impact of interaction of vitamin D and gut microbiota on benefit clinical radiotherapy outcomes in patients with cancer are warranted.

## Data Availability

Data sharing not applicable to this article as no datasets were generated or analysed during the current study.

## References

[1] Lin L, Yan L, Liu Y, Yuan F, Li H, Ni J (2019). Incidence and death in 29 cancer groups in 2017 and trend analysis from 1990 to 2017 from the global burden of disease study. J Hematol Oncol.

[2] Ferlay J, Colombet M, Soerjomataram I, Mathers C, Parkin DM, Pineros M, Znaor A, Bray F (2019). Estimating the global cancer incidence and mortality in 2018: GLOBOCAN sources and methods. Int J Cancer.

[3] Veness MJ, Delishaj D, Barnes EA, Bezugly A, Rembielak A. Current role of radiotherapy in non-melanoma skin Cancer. Clin Oncol. 2019;31(11):749–58. 10.1016/j.clon.2019.08.004 31447088

[4] Taylor C, Correa C, Duane FK, Aznar MC, Anderson SJ, Bergh J, Dodwell D, Ewertz M, Gray R, Jagsi R (2017). Estimating the risks of breast Cancer radiotherapy: evidence from modern radiation doses to the lungs and heart and from previous randomized trials. J Clin Oncol : Official J Am Society Clin Oncol.

[5] Ma S, Zhang T, Jiang L, Qin W, Lu K, Zhang Y, Wang R (2019). Impact of bladder volume on treatment planning and clinical outcomes of radiotherapy for patients with cervical cancer. Cancer Manag Res.

[6] Stone L (2019). DDR pathway mediates radiosensitization in prostate cancer. Nature Rev Urol.

[7] Biau J, Chautard E, De Koning L, Court F, Pereira B, Verrelle P, Dutreix M (2017). Predictive biomarkers of resistance to hypofractionated radiotherapy in high grade glioma. Radiat Oncol.

[8] Huang S, Zhan Z, Li L, Guo H, Yao Y, Feng M, Deng J, Xiong J (2019). LINC00958-MYC positive feedback loop modulates resistance of head and neck squamous cell carcinoma cells to chemo- and radiotherapy in vitro. OncoTargets Ther.

[9] Huang R, Zhou Y, Hu S, Ren G, Cui F, Zhou PK (2019). Radiotherapy exposure in Cancer patients and subsequent risk of stroke: a systematic review and meta-analysis. Front Neurol.

[10] Huang R, Yu T, Li Y, Hu J (2018). Upregulated has-miR-4516 as a potential biomarker for early diagnosis of dust-induced pulmonary fibrosis in patients with pneumoconiosis. Toxicol Res.

[11] Qin J, Ning H, Zhou Y, Hu Y, Yang L, Huang R (2018). LncRNA MIR31HG overexpression serves as poor prognostic biomarker and promotes cells proliferation in lung adenocarcinoma. Biomedicine & Pharmacotherapy = Biomedecine & Pharmacotherapie.

[12] Liu XD, Xie DF, Wang YL, Guan H, Huang RX, Zhou PK (2019). Integrated analysis of lncRNA-mRNA co-expression networks in the alpha-particle induced carcinogenesis of human branchial epithelial cells. Int J Radiat Biol.

[13] Zhou PK, Huang RX (2018). Targeting of the respiratory chain by toxicants: beyond the toxicities to mitochondrial morphology. Toxicol Res (Camb).

[14] Castro-Eguiluz D, Leyva-Islas JA, Luvian-Morales J, Martinez-Roque V, Sanchez-Lopez M, Trejo-Duran G, Jimenez-Lima R, Leyva-Rendon FJ (2018). Nutrient Recommendations for Cancer Patients Treated with Pelvic Radiotherapy, with or without Comorbidities. Revista de investigacion clinica; organo del Hospital de Enfermedades de la Nutricion.

[15] Wei L, Wang X, Lv L, Liu J, Xing H, Song Y, Xie M, Lei T, Zhang N, Yang M (2019). The emerging role of microRNAs and long noncoding RNAs in drug resistance of hepatocellular carcinoma. Mol Cancer.

[16] Shuryak I (2019). Review of microbial resistance to chronic ionizing radiation exposure under environmental conditions. J Environ Radioact.

[17] Mo LJ, Song M, Huang QH, Guan H, Liu XD, Xie DF, Huang B, Huang RX, Zhou PK (2018). Exosome-packaged miR-1246 contributes to bystander DNA damage by targeting LIG4. Br J Cancer.

[18] Bai J, Guo XG, Bai XP (2012). Epidermal growth factor receptor-related DNA repair and radiation-resistance regulatory mechanisms: a mini-review. Asian Pacific J Cancer Prev : APJCP.

[19] Yuan Y, Zhang Y, Han L, Sun S, Shu Y (2018). miR-183 inhibits autophagy and apoptosis in gastric cancer cells by targeting ultraviolet radiation resistance-associated gene. Int J Mol Med.

[20] Chen N, Wu L, Yuan H, Wang J (2015). ROS/autophagy/Nrf2 pathway mediated low-dose radiation induced radio-resistance in human lung adenocarcinoma A549 cell. Int J Biol Sci.

[21] Kim BM, Hong Y, Lee S, Liu P, Lim JH, Lee YH, Lee TH, Chang KT, Hong Y (2015). Therapeutic implications for overcoming radiation resistance in Cancer therapy. Int J Mol Sci.

[22] Zheng K, He Z, Kitazato K, Wang Y (2019). Selective autophagy regulates cell cycle in Cancer therapy. Theranostics.

[23] Sampaio-Marques B, Guedes A, Vasilevskiy I, Goncalves S, Outeiro TF, Winderickx J, Burhans WC, Ludovico P (2019). Alpha-Synuclein toxicity in yeast and human cells is caused by cell cycle re-entry and autophagy degradation of ribonucleotide reductase 1. Aging Cell.

[24] Huang P, Sun LY, Zhang YQ (2019). A hopeful natural product, Pristimerin, induces apoptosis, cell cycle arrest, and autophagy in esophageal Cancer cells. Anal Cell Pathol.

[25] Rice MW, Pandya JD, Shear DA (2019). Gut microbiota as a therapeutic target to ameliorate the biochemical, neuroanatomical, and behavioral effects of traumatic brain injuries. Front Neurol.

[26] Villanueva-Millan MJ, Perez-Matute P, Oteo JA (2015). Gut microbiota: a key player in health and disease. A review focused on obesity. J Physiol Biochem.

[27] Touchefeu Y, Montassier E, Nieman K, Gastinne T, Potel G, Bruley des Varannes S, Le Vacon F, de La Cochetiere MF (2014). Systematic review: the role of the gut microbiota in chemotherapy- or radiation-induced gastrointestinal mucositis - current evidence and potential clinical applications. Aliment Pharmacol Ther.

[28] Alexander JL, Wilson ID, Teare J, Marchesi JR, Nicholson JK, Kinross JM (2017). Gut microbiota modulation of chemotherapy efficacy and toxicity. Nat Rev Gastroenterol Hepatol.

[29] Ma W, Mao Q, Xia W, Dong G, Yu C, Jiang F (2019). Gut microbiota shapes the efficiency of Cancer therapy. Front Microbiol.

[30] Tilg H, Zmora N, Adolph TE, Elinav E. The intestinal microbiota fuelling metabolic inflammation. Nat Rev Immunol. 2019. 10.1038/s41577-019-0198-4 10.1038/s41577-019-0198-4 31388093

[31] Ghaly S, Kaakoush NO, Lloyd F, Gordon L, Forest C, Lawrance IC, Hart PH. Ultraviolet Irradiation of Skin Alters the Faecal Microbiome Independently of Vitamin D in Mice. Nutrients. 2018;10(8). 10.3390/nu10081069 PMC6116187 30103486

[32] Barker HE, Paget JT, Khan AA, Harrington KJ (2015). The tumour microenvironment after radiotherapy: mechanisms of resistance and recurrence. Nat Rev Cancer.

[33] Gosiewski T, Mroz T, Ochonska D, Pabian W, Bulanda M, Brzychczy-Wloch M (2016). A study of the effects of therapeutic doses of ionizing radiation in vitro on Lactobacillus isolates originating from the vagina - a pilot study. BMC Microbiol.

[34] Dima C, Dima S (2019). Bioaccessibility study of calcium and vitamin D3 co-microencapsulated in water-in-oil-in-water double emulsions. Food Chem.

[35] Bae YJ, Kratzsch J (2018). Vitamin D and calcium in the human breast milk. Best Pract Res Clin Endocrinol Metab.

[36] Harinarayan CV, Akhila H (2019). Modern India and the tale of twin nutrient deficiency-calcium and vitamin D-nutrition trend data 50 years-retrospect, introspect, and Prospect. Front Endocrinol.

[37] Abrahamsson H, Porojnicu AC, Lindstrom JC, Dueland S, Flatmark K, Hole KH, Seierstad T, Moan J, Redalen KR, Meltzer S (2019). High level of circulating vitamin D during neoadjuvant therapy may lower risk of metastatic progression in high-risk rectal cancer. BMC Cancer.

[38] van den Blink QU, Garcez K, Henson CC, Davidson SE, Higham CE (2018). Pharmacological interventions for the prevention of insufficiency fractures and avascular necrosis associated with pelvic radiotherapy in adults. The Cochrane Database of Systematic Reviews.

[39] Christakos S, Dhawan P, Verstuyf A, Verlinden L, Carmeliet G (2016). Vitamin D: metabolism, molecular mechanism of action, and pleiotropic effects. Physiol Rev.

[40] Liu KH, Fu J, Zhou N, Yin W, Yang YY, Ouyang SX, Liang YM (2019). 1,25-Dihydroxyvitamin D3 prevents epithelial-Mesenchymal transition of HMrSV5 human peritoneal Mesothelial cells by inhibiting histone Deacetylase 3 (HDAC3) and increasing vitamin D receptor (VDR) expression through the Wnt/beta-catenin signaling pathway. Med Sci Monit : Int Med J Exp Clin Res.

[41] Hou YF, Gao SH, Wang P, Zhang HM, Liu LZ, Ye MX, Zhou GM, Zhang ZL, Li BY. 1alpha,25(OH)(2)D(3) Suppresses the Migration of Ovarian Cancer SKOV-3 Cells through the Inhibition of Epithelial-Mesenchymal Transition. Int J Mol Sci. 2016;17(8). 10.3390/ijms17081285 PMC5000682 27548154

[42] Findlay VJ, Moretz RE, Wang C, Vaena SG, Bandurraga SG, Ashenafi M, Marshall DT, Watson DK, Camp ER (2014). Slug expression inhibits calcitriol-mediated sensitivity to radiation in colorectal cancer. Mol Carcinog.

[43] Ramakrishnan S, Steck SE, Arab L, Zhang H, Bensen JT, Fontham ETH, Johnson CS, Mohler JL, Smith GJ, Su LJ (2019). Association among plasma 1,25(OH)2 D, ratio of 1,25(OH)2 D to 25(OH)D, and prostate cancer aggressiveness. Prostate.

[44] Al Mutair AN, Nasrat GH, Russell DW (2012). Mutation of the CYP2R1 vitamin D 25-hydroxylase in a Saudi Arabian family with severe vitamin D deficiency. J Clin Endocrinol Metab.

[45] Aranow C (2011). Vitamin D and the immune system. J Invest Med : The Official Publication Am Federation Clin Res.

[46] Lee Y, Yun SJ, Kim Y, Kim MS, Han GH, Sood AK, Kim J (2017). Near-field spectral mapping of individual exciton complexes of monolayer WS2 correlated with local defects and charge population. Nanoscale.

[47] Skaaby T, Thuesen BH, Linneberg A (2017). Vitamin D, cardiovascular disease and risk factors. Adv Exp Med Biol.

[48] Pilz S, Verheyen N, Grubler MR, Tomaschitz A, Marz W (2016). Vitamin D and cardiovascular disease prevention. Nat Rev Cardiol.

[49] Mukai Y, Yamada D, Eguchi H, Iwagami Y, Asaoka T, Noda T, Kawamoto K, Gotoh K, Kobayashi S, Takeda Y (2018). Vitamin D supplementation is a promising therapy for pancreatic ductal adenocarcinoma in conjunction with current Chemoradiation therapy. Ann Surg Oncol.

[50] Nasser NJ, Fenig S, Ravid A, Nouriel A, Ozery N, Gardyn S, Koren R, Fenig E (2017). Vitamin D ointment for prevention of radiation dermatitis in breast cancer patients. NPJ Breast Cancer.

[51] Branca JJ, Pacini S, Ruggiero M (2015). Effects of pre-surgical vitamin D supplementation and Ketogenic diet in a patient with recurrent breast Cancer. Anticancer Res.

[52] Ghorbanzadeh-Moghaddam A, Gholamrezaei A, Hemati S (2015). Vitamin D deficiency is associated with the severity of radiation-induced Proctitis in Cancer patients. Int J Radiat Oncol Biol Phys.

[53] Skrajnowska D, Bobrowska-Korczak B (2019). Potential molecular mechanisms of the anti-cancer activity of vitamin D. Anticancer Res.

[54] Sharma K, Goehe RW, Di X, Hicks MA, Torti SV, Torti FM, Harada H, Gewirtz DA (2014). A novel cytostatic form of autophagy in sensitization of non-small cell lung cancer cells to radiation by vitamin D and the vitamin D analog, EB 1089. Autophagy.

[55] Wilson EN, Bristol ML, Di X, Maltese WA, Koterba K, Beckman MJ, Gewirtz DA (2011). A switch between cytoprotective and cytotoxic autophagy in the radiosensitization of breast tumor cells by chloroquine and vitamin D. Hormones & cancer.

[56] Gonzalo S (2014). Novel roles of 1alpha,25(OH)2D3 on DNA repair provide new strategies for breast cancer treatment. The Journal of Steroid Biochemistry and Molecular Biology.

[57] Barbachano A, Fernandez-Barral A, Ferrer-Mayorga G, Costales-Carrera A, Larriba MJ, Munoz A (2017). The endocrine vitamin D system in the gut. Mol Cell Endocrinol.

[58] Sun J (2016). VDR/vitamin D receptor regulates autophagic activity through ATG16L1. Autophagy.

[59] Bashir M, Prietl B, Tauschmann M, Mautner SI, Kump PK, Treiber G, Wurm P, Gorkiewicz G, Hogenauer C, Pieber TR (2016). Effects of high doses of vitamin D3 on mucosa-associated gut microbiome vary between regions of the human gastrointestinal tract. Eur J Nutr.

[60] Kanhere M, Chassaing B, Gewirtz AT, Tangpricha V (2018). Role of vitamin D on gut microbiota in cystic fibrosis. J Steroid Biochem Mol Biol.

[61] Gubatan J, Moss AC (2018). Vitamin D in inflammatory bowel disease: more than just a supplement. Curr Opin Gastroenterol.

[62] Cantorna MT, Snyder L, Arora J (2019). Vitamin a and vitamin D regulate the microbial complexity, barrier function, and the mucosal immune responses to ensure intestinal homeostasis. Crit Rev Biochem Mol Biol.

[63] Mandle HB, Jahan FA, Bostick RM, Baron JA, Barry EL, Yacoub R, Merrill J, Rutherford RE, Seabrook ME, Fedirko V (2019). Effects of supplemental calcium and vitamin D on tight-junction proteins and mucin-12 expression in the normal rectal mucosa of colorectal adenoma patients. Mol Carcinog.

[64] Zhu W, Yan J, Zhi C, Zhou Q, Yuan X (2019). 1,25(OH)2D3 Deficiency-induced gut microbial dysbiosis degrades the colonic mucus barrier in Cyp27b1 knockout mouse model. Gut Pathogens.

[65] Paul S, Smilenov LB, Elliston CD, Amundson SA (2015). Radiation dose-rate effects on gene expression in a mouse Biodosimetry model. Radiat Res.

[66] Kirischian NL, Wilson JY (2012). Phylogenetic and functional analyses of the cytochrome P450 family 4. Mol Phylogenet Evol.

[67] Goudarzi M, Chauthe S, Strawn SJ, Weber WM, Brenner DJ, Fornace AJ. Quantitative Metabolomic Analysis of Urinary Citrulline and Calcitroic Acid in Mice after Exposure to Various Types of Ionizing Radiation. Int J Mol Sci. 2016;17(5). 10.3390/ijms17050782 PMC4881599 27213362

[68] Ferrer-Mayorga G, Larriba MJ, Crespo P, Munoz A (2019). Mechanisms of action of vitamin D in colon cancer. J Steroid Biochem Mol Biol.

[69] Singh P, Kumar M, Al Khodor S (2019). Vitamin D deficiency in the Gulf cooperation council: exploring the triad of genetic predisposition, the gut microbiome and the immune system. Front Immunol.

[70] Zhang Z, Thorne JL, Moore JB (2019). Vitamin D and nonalcoholic fatty liver disease. Curr Opin Clin Nutr Metab Care.

[71] Tabatabaeizadeh SA, Tafazoli N, Ferns GA, Avan A, Ghayour-Mobarhan M (2018). Vitamin D, the gut microbiome and inflammatory bowel disease. J Res Med Sci : Official J Isfahan University Med Sci.

[72] Waterhouse M, Hope B, Krause L, Morrison M, Protani MM, Zakrzewski M, Neale RE (2019). Vitamin D and the gut microbiome: a systematic review of in vivo studies. Eur J Nutr.

[73] Wang J, Thingholm LB, Skieceviciene J, Rausch P, Kummen M, Hov JR, Degenhardt F, Heinsen FA, Ruhlemann MC, Szymczak S (2016). Genome-wide association analysis identifies variation in vitamin D receptor and other host factors influencing the gut microbiota. Nat Genet.

[74] Bakke D, Sun J (2018). Ancient nuclear receptor VDR with new functions: microbiome and inflammation. Inflamm Bowel Dis.

[75] Su D, Nie Y, Zhu A, Chen Z, Wu P, Zhang L, Luo M, Sun Q, Cai L, Lai Y (2016). Vitamin D signaling through induction of Paneth cell Defensins maintains gut microbiota and improves metabolic disorders and hepatic Steatosis in animal models. Front Physiol.

[76] He JY, Wang WZ, Qi HZ, Ma Y, He SY (2018). Use of recombinant Lactobacillus sakei for the prevention and treatment of radiation-induced enteritis. Med Hypotheses.

[77] Nascimento M, Caporossi C, Aguilar-Nascimento JE, Castro-Barcellos HM, Motta RT, Lima SR. Efficacy of Synbiotics to reduce symptoms and rectal inflammatory response in acute radiation Proctitis: a randomized, double-blind, placebo-controlled pilot trial. Nutr Cancer. 2019:1–8. 10.1080/01635581.2019.1647254 10.1080/01635581.2019.1647254 31364875

[78] Fox JG, Ge Z, Whary MT, Erdman SE, Horwitz BH (2011). Helicobacter hepaticus infection in mice: models for understanding lower bowel inflammation and cancer. Mucosal Immunol.

[79] Ottman N, Reunanen J, Meijerink M, Pietila TE, Kainulainen V, Klievink J, Huuskonen L, Aalvink S, Skurnik M, Boeren S (2017). Pili-like proteins of Akkermansia muciniphila modulate host immune responses and gut barrier function. PLos One.

[80] Bora SA, Kennett MJ, Smith PB, Patterson AD, Cantorna MT (2018). The gut microbiota regulates endocrine vitamin D metabolism through fibroblast growth factor 23. Front Immunol.

[81] Bora SA, Kennett MJ, Smith PB, Patterson AD, Cantorna MT (2018). Regulation of vitamin D metabolism following disruption of the microbiota using broad spectrum antibiotics. J Nutr Biochem.

[82] Zuo K, Li J, Xu Q, Hu C, Gao Y, Chen M, Hu R, Liu Y, Chi H, Yin Q (2019). Dysbiotic gut microbes may contribute to hypertension by limiting vitamin D production. Clin Cardiol.

